# Sedentary behaviour levels in adults with an intellectual disability: a systematic review and meta-analysis

**DOI:** 10.12688/hrbopenres.13326.3

**Published:** 2022-03-30

**Authors:** Louise Lynch, Mary McCarron, Philip McCallion, Eilish Burke

**Affiliations:** 1 School of Nursing and Midwifery, Trinity College, Dublin, Dublin, Ireland; 2 School of Social work, College of Public Health, Temple University, Philadelphia, Pennsylvania, USA

**Keywords:** Intellectual disability, sedentary behaviour, adults

## Abstract

**Background**: Sedentary behaviour (SB), which is characterised by low levels of energy expenditure, has been linked to increased cardio-metabolic risks, obesity and mortality, as well as cancer risk. No firm guidelines are established on safe levels of SB. Adults with an intellectual disability (ID) have poorer health than their counterparts in the general population with higher rates of multi-morbidity, inactivity, and obesity. The reasons for this health disparity are unclear however it is known that SB and overall inactivity contribute to poorer health. There is no clear picture of the levels of SB among individuals with ID therefore SB levels in this vulnerable population need to be examined. The aim of this systematic review is to investigate the prevalence of sedentary behaviour in adults with an ID.

**Methods**: The PRISMA-P framework was applied to identify high quality articles. An extensive search was carried out in four databases and grey literature sources . In total, 1,972 articles were retrieved of which 48 articles went forward for full review after duplicate removal and screening by title and abstract. The National Institute of Health’s quality assessment tools were used to assess article quality. Two reviewers independently assessed each article. An excel spreadsheet was created to guide the data extraction process. The final review included 25 articles. A meta-analysis was completed using REVMAN.

**Results**: Different SB assessment types were identified in studies. These included steps, time, questionnaires, and screen time. Studies were heterogeneous. Observed daily steps per individual ranged from 44 to above 30,000, with an average of approximately 6,500 steps. Mean daily time spent in SBs was more than 60% of available time, with observed screen time of more than 3 hours.

**Conclusion**: There is a high prevalence of SB in adults with an intellectual disability.

[Registration no: Index CRD42020177225].

## 1.0 Introduction

Intellectual disability (ID) begins before adulthood and is defined as having an impaired intelligence which results in impaired social functioning, with a lasting effect on development ( WHO, 2020a). In 2016 approximately 1.4% of the Irish population, were shown to have an ID, the equivalent of over 70,000 people ( Census, 2016). Worldwide people with an ID constitute approximately 1% of the population ( Maulik *et al.*, 2011).

People, including those with an ID now live longer than they did in previous decades ( McCarron *et al.*, 2015). Therefore, a need exists to facilitate healthy aging and prevent age-related diseases. One factor that contributes to a longer, healthy lifestyle is being physically active. However, 25% of the world’s adult population do not meet recommendations for activity levels and Ireland’s older population is one of the most inactive in Europe ( Bartlo & Klein, 2011; Loyen *et al.*, 2016; WHO, 2020b). Inactivity contributes to all-cause mortality ( WHO, 2020b). Low levels of activity are associated with poorer health outcomes and in a recent study by Tyrer and colleagues (2019), inactivity was associated with higher rates of multi-morbidity. Older people with ID have been shown to have higher rates of multi-morbidity, obesity, and inactivity than the general population ( Gawlik *et al.*, 2018; McCarron *et al.*, 2013; Tyrer *et al.*, 2019). Often their health experience is poorer than their non-disabled peers with a higher prevalence of health disparity ( Emerson *et al.*, 2016; Krahn & Fox, 2014). According to Graham & Reid (2000), adults with ID are more susceptible to age-related health risks. Another study with people with ID identified obesity levels, a major factor underpinning many health conditions, ranging from 28%–71%, where SB was one of the main contributors ( Ranjan *et al.*, 2018). This poorer health status increases individual’s risk of greater use of healthcare services and consequent higher healthcare costs. In the US over $51 billion was attributed to healthcare costs of those with ID, which equated to over three times the cost of an individual from the general population ( Catlin & Cowan, 2015; Honeycutt *et al.*, 2003). However, this poorer health status can be ameliorated through a multifactorial lifestyle approach, one aspect being the promotion of increased movement ( Fock & Khoo, 2013). Considering that individuals with ID have higher levels of ill health, die nearly 20 years earlier than their peers in the general population and are noted as being more inactive, their risk of ageing in poorer health is increased ( Krahn *et al.*, 2006; Krahn & Fox, 2014; McCarron *et al.*, 2015). This can be attributed to disparity in health and avoidable causes of poor health such as type 2 diabetes, which are amenable to change through the introduction of improved lifestyle particularly with the introduction of physical activity ( O’Leary *et al.*, 2018). However, for individuals with ID managing their own health poses challenges ( Burke *et al.*, 2017). A better understanding of SB is necessary, to inform policy makers to facilitate change for this vulnerable population.

In general, individuals with ID have lower physical activity (PA) levels than the general population and this is a potential contributor to poorer health in this group ( Burke *et al.*, 2017). Using self-reported methods, Wave 3 of The Intellectual Disability Supplement to The Irish Longitudinal Study on Aging (IDS-TILDA), identified that more than 70% of participants were inactive ( Burke *et al.*, 2017). Similarly, Marconi *et al.* (2018) and, Phillips & Holland (2011) found that individuals with ID did not attain the recommended daily PA levels and what is of concern is that levels declined notably as they aged. Similarly, a recent Australian based study found that over 66% of participants with ID did not meet minimum exercise guidelines ( Koritsas & Iacono, 2016), while another US study found 77% of participants did not meet minimum exercise recommendations ( Barnes *et al.*, 2013). Hence inactivity and particularly sedentary behaviour is a global problem.

### 1.1. Sedentary behaviour (SB)

Sedentary Behaviour (SB) and physical inactivity are frequently seen as one and the same, however they are very different and should be addressed separately. While recommendations for movement and PA levels in adults are long established for health benefits, corresponding recommended levels for time spent in SB, other than to reduce SB, are not ( Bull *et al.*, 2020).

In an effort to provide clarity, in the literature, SB has been defined as ‘any waking behaviour characterized by an energy expenditure of ≤1.5 METs while in a sitting, lying or reclining posture’ for example watching television or working on a computer ( Tremblay *et al.*, 2017, p. 9). Hence SB constitutes too much sitting or stationary activity as opposed to physical inactivity which is too little exercise or physical movement. A scoping review revealed that many publications have confused physical inactivity and sedentary behaviour. Hence a much broader definition of SB was refined for the purposes of this systematic review to also include physical inactivity and thus support the thorough identification of the prevalence of SB among this population and capture all relevant, seminal pieces. The definition of SB for the purposes of this systematic review is:

‘Low physical activity as identified by metabolic equivalent (MET) or step levels or as measured by the Rapid Assessment of Physical activity questionnaire (RAPA) or the International Physical Activity questionnaire (IPAQ) or sitting, lying or reclining for more than 3 hours per day’.

A metabolic equivalent (MET), known as the resting metabolic rate, is an objective measurement scale used to classify activity types and levels. A MET is the amount of oxygen (O _2_) burned at rest and is the equivalent of 3.5ml O _2_ per kg bodyweight per minute ( Jette *et al.*, 1990) or 1kilocalorie per kg of bodyweight per hour ( Newton *et al.*, 2013).

In the general population, time spent in SB has been linked to increased cardio-metabolic risks, increased obesity and mortality, as well as increased cancer risk ( de Rezende *et al.*, 2014; Patel *et al.*, 2010; Same *et al.*, 2016; Thorp *et al.*, 2011). Emerging evidence is highlighting the importance of reducing SB time for improving cardio-metabolic health. The same body of evidence is supporting the adoption of a holistic public health approach to improving activity levels as well as reducing SB time ( van der Ploeg & Hillsdon, 2017). High levels of SB, even if minimum exercise guidelines are met, show increased risk of heart disease, diabetes, and stroke ( Patel *et al.*, 2010).

However, the detrimental impact of SB can be reduced by interspersing periods of PA throughout the day ( Healy *et al.*, 2008). While breaking up time spent being sedentary has been shown to improve physical performance in everyday activities in older adults ( Sardinha *et al.*, 2015), there is no similar information on adults with ID. This systematic review was conducted to explore the state of the science of sedentary behaviour in adults with an intellectual disability. It is critical that this information is identified so that they may be supported to age in a positive way. Overall, the effects of time spent in SB is poorly understood. The aim of this systematic review is to understand the prevalence of SB in adults with an intellectual disability.

## 2.0. Methods

This systematic literature review was designed to understand the prevalence of time spent in sedentary behaviour (SB) in the adult ID community. The researcher has written, registered with Prospero and published the systematic review protocol [Index CRD42020177225]. PRISMA-P, for the reporting and development of systematic review protocols was used as the guide for the writing of this protocol ( Shamseer *et al.*, 2015). The full methodology details for this systematic review are available in the protocol ( Lynch *et al.*, 2021). However, a synopsis is provided here.

### 2.1. Research question

PICO, which is used for quantitative studies was used to define the question as follows ( Schardt *et al.*, 2007):

- P [Population or problem]: Adults aged 18+ with an Intellectual Disability
- I [Intervention or exposure]: Sedentary behaviour level (SB in line with the definition of SB defined for this review
- C [Comparison]: Individuals with all levels of ID living in residential, institutional or hospital settings, community group homes, with family or independently
- O [Outcome]: Prevalence of Sedentary behaviour

The research question to be addressed is:

*‘What are the sedentary behaviour levels of Adults with an Intellectual Disability?’.*

### 2.2. Eligibility criteria

The criteria for study inclusion in the review are as follows:

- Population: adults aged 18+ with an Intellectual Disability
- Language: English
- Study type: All types of studies including primary studies, peer reviewed, grey literature
- Study design: Randomised controlled trials, cohort, cross-sectional
- Content: Must reference sedentary behaviours of adults with ID to be eligible for inclusion
- Timeframe: no restriction on timeframes up to March 2020.

The criteria for exclusion in the review are as follows:

- Population: Children with or without an ID and Adults without ID
- Language: Articles that are not available in English
- Study design: Any type of reviews
- Conference proceedings and published conference abstracts only

### 2.3 Information sources

***2.3.1. Databases*.** The following four databases were used to perform the search:

- Medline
- Embase
- psycINFO
- Cinahl

In addition, the following sources were explored for grey literature sources:

- The CORDIS library
- Grey Literature Database from the Canadian Evaluation Society
- The U.S. Department of Housing and Urban Development (HUD) User database
- National Technical Information Service (NTIS)
- Open Grey
- Social Care Online
- Social Science Research Network (SSRN) eLibrary
- RIAN
- Google Scholar
- Proquest (Dissertations and Theses)

***2.3.2 Search strategy*.** The search strategy was refined into two concepts following the application of PICO. Concept 1 is ‘Sedentary behaviour or inactivity’ and Concept 2 is ‘Intellectual Disability’. Each of the two concepts were searched using MESH terms and keywords and then combined using OR. Then the total results of each concept were combined using AND. A figure representing the search strategy is available in extended data ( Lynch, 2021). This search was repeated for each of the four databases. The resulting article list was the complete combined database search results. This list was screened for inclusion.

***Search string*.** An example of the search string used for the Medline database is shown in Table 1.

**Table 1.** Medline search string.

Concept	Index	Keywords
**Concept 1:** Sedentary behaviour & physical inactivity	(MH "Sedentary Behavior")	sedentary lifestyle* OR sedentary behavior* OR sedentary behaviour* OR physical* inactiv* OR inactive lifestyle
**Concept 2:** Intellectual disability or learning disability	(MH "Intellectual Disability+") OR (MH "Learning Disabilities+")	((intellectual AND disabilit* OR 'mental retardation'/exp OR 'mental retardation' OR (mental AND ('retardation'/exp OR retardation)) OR 'learning'/ exp OR learning) AND disabilit* OR developmental) AND disabilit* OR 'learning disabilities'/exp OR 'learning disabilities' OR (('learning'/exp OR learning) AND disabilities)

***2.3.3. Screening process.*** All identified articles from each database that is searched, as well as all grey literature sources, were combined and duplicates removed. Endnote software was used to store all the identified articles. The articles were stored in folders which were named after the search process used. Using the inclusion criteria as detailed above, all articles were initially screened by title and then by abstract. The remaining full text articles were retrieved and read thoroughly. Those that did not meet the inclusion criteria were omitted.

### 2.4 Quality assessment and risk of bias

The remaining articles were quality assessed by two separate assessors using two validated quality assessment tools from the National Institute of Health (NIH) ( National Institute Health, 2020), the first for observational cohort and cross-sectional studies and the second for randomised controlled trials (RCTs). A third person was available as an adjudicator for any discrepancies. The tools used are available as extended data ( Lynch, 2021).

There are different types of study quality assessment tools for the different study types. For Controlled Intervention Studies and Observational Cohort and Cross-sectional studies, 14 criteria were used to evaluate the study quality, while for Case-Control studies 12 criteria were used. 11 criteria were used to determine the study quality of RCTs. This means that a maximum quality score of 11, 12 or14 could be achieved depending on the study type. This quality score was used to determine if the study should be included in the review. Quality scores were divided into 3 main categories: Good, Fair or Poor. See Table 2 for details.

**Table 2.** Quality assessment Scoring System.

Quality Rating	Observational Cohort & Cross-Sectional Studies	Case Control Studies	RCTs	Action
Good	9–12	10–14	7–11	Data extraction
Fair	6–8	7–9	4–6	2 reviewers to discuss. Adjudicate with 3rd reviewer if required.
Poor	<=5	<=6	<=4	2 reviewers to discuss. Reject
Other	CD, NR, NA*			

***2.4.1. Quality scoring*.** Scores were attributed to distinct parts of the study design for example type of study, design and blinding, where a ‘yes’ answer gives a score of ‘1’, a ‘no’ answer a score of ‘0’ and could potentially highlight an issue with the article.

## 3.0 Findings

### 3.1. Screening Process

**Figure 1.**
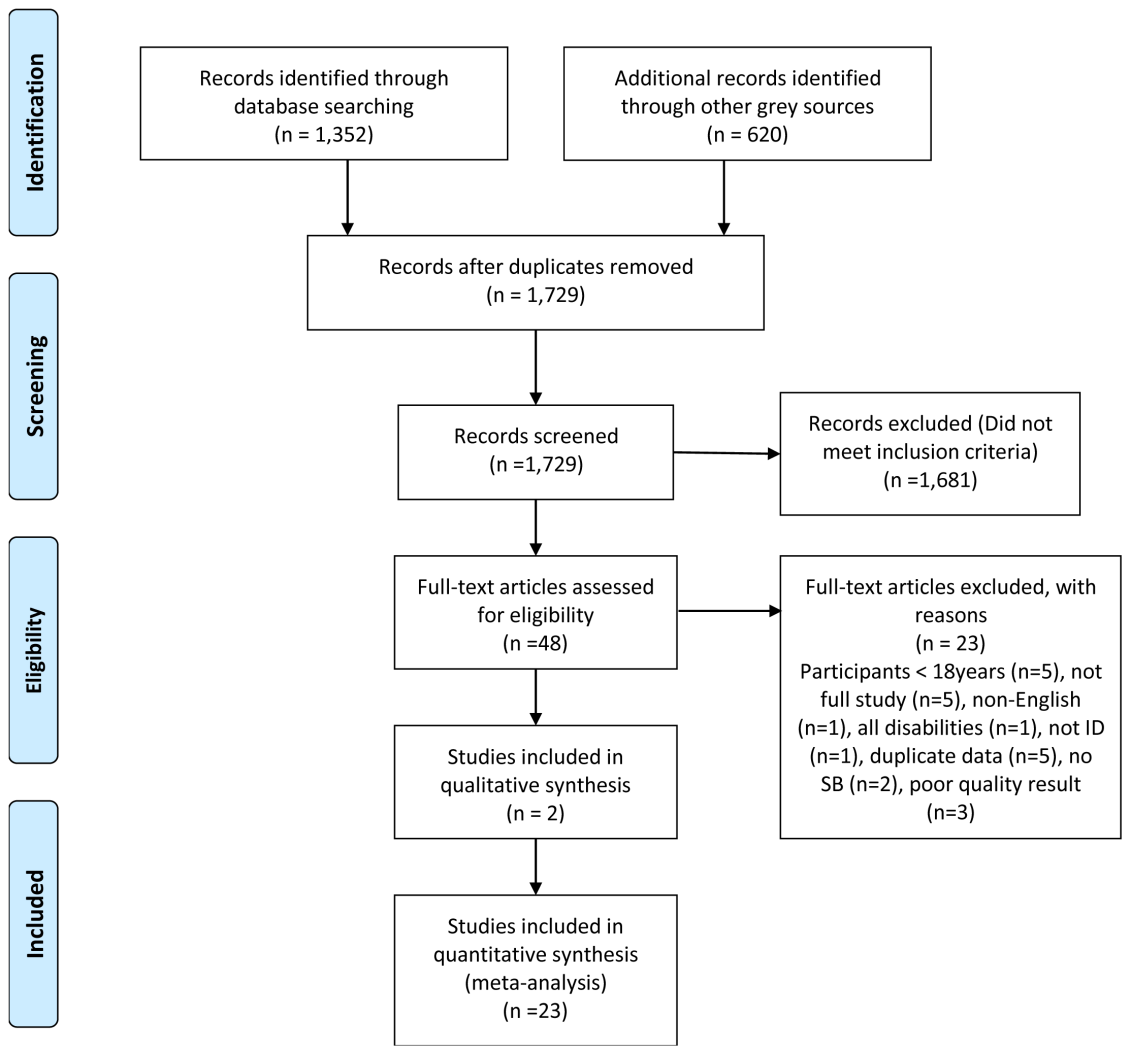
PRISMA search flowchart.

The PRISMA search flowchart is shown in Figure 1.

An excel spreadsheet served as the data extraction tool to summarise the remaining articles. Article details were captured under 25 category headings. Exclusion criteria eliminated 20 articles. Two assessors [LL, EB] reviewed and quality assessed each of the final articles. There were no big discrepancies in results so a third adjudicator [MMcC] was not required.

### 3.2. Quality assessment and risk of bias

The final number of articles that went forward for a full quality assessment was 28. Using the NIH’s quality assessment tools for observational, cohort and cross-sectional studies and Randomised Control Studies (RCTs) to assess the internal validity of each article and any sources of potential bias, ( National Institute Health, 2020). only articles rated in the fair to good range by the two assessors [LL and EB] were included. Appropriate quality scores for inclusion in this systematic review were achieved by 25 articles. These 25 articles are summarised in Table 3.

**Table 3.** Final articles used.

No	Article	No of Parts.	Study Focus	Assessment type	Measurement device	Sedentary or (in)activity	Country	Age	Gender	Level of ID
1	Temple & Walkley, 2003.	37	Concurrence of accelerometer readings of PA and proxy generated estimates of PA via diary recordings	Staff completed Bouchard 3day activity record (which has 9 point scale) and accelerometer worn for the 3 days of this diary completion	Caltrac accelerometer	PA	Australia	N/A	18 women, 19 men	Mild to Mod
2	Temple, 2007.	37	Examine relationships between doing PA/sedentary behaviour & factors consistent with behavioural choice theory enjoyment, preference and barriers	Pedometer	Yamax Digi walker SW-700	PA	USA	18-52 yrs.	18 men, 19 women	Not specified
3	Peterson *et al*., 2008.	131	Aim to objectively monitor steps accrued by sample of 131 adults with ID and describe patterns across day and week	Steps via pedometer	Omron pedometer HJ-700IT, worn for 7 days	PA	US (IOWA)	Mean age of 37.2 yrs (18 -60 yrs)	51.9% female	73 mild, 41 mod, 17 unknown, 28 DS
4	Finlayson *et al*., 2011.	62. 41 worn activity monitor for at least 5 days	Aim of study is to measure the levels and patterns of activity of adults with ID to inform design of studies aimed at increasing activity and health in this population	Self-report and activity monitor	ActivPal. Interview before and after 7 day period	Both	UK, Scotland	Mean age 37.1 (Range 18-66)	27 males, 35 females	Mild to mod
5	Matthews *et al*., 2011.	45	To assess level of agreement between accelerometer and proxy- respondent questionnaire (IPAQ short version). Obese focus	IPAQ and accelerometer. Wore device for 7 consecutive days. Kept diary of non-wear times	Actigraph GT1M	Both	Scotland, UK	Mean age = 48.3 years (23-72)	17 male, 28 female	30 mild/mod 15 severe
6	Hilgenkamp *et al*., 2012.	257 (out of group of 1050)	Measure the physical activity levels of older (50+) adults with ID	Pedometer. Caregiver was instructed to record the no of steps, distance and activity in minutes in a diary every evening. Worn for 14 days	NL-1000 pedometer. Said to take reliable measurements at walking speed of >= 3.2km/hr	PA	Holland	50+ years. 50-59: 146 60-69:83, 70- 79:25, 80-89:3	133 men, 124 female	Borderline-11, mild-88, mod-143, severe-10, prof-0, unknown -5
7	Bergström *et al*., 2013.	130	To improve diet and PA by a 3 way complex approach using Social Cognitive Theory, targeting caregivers and residences	Average No of steps per day (3 valid days min). Results of 500 steps per day or less were discounted as being inaccurate. Steps taken each day were recorded each evening.	Keep walking LS2000	PA (by pedometry)	Sweden	20-66 years	74 women, 56 men	Mild & mod
8	Dixon-Ibarra *et al*., 2013.	109	To examine the physical activity patterns of older adults with ID compared with younger adults with ID and older adults without ID	Pedometers and accelerometers	GT1M actigraph accelerometer and Omron HJ 720ITC Pedometer	Both. Sed classed as when accelerometer registers <100 counts/min	USA	Mean age adults w/ID= 32.34 mean age older adults w/ID= 57.87	48 = male, 36 = female.	Mild to mod ID. 20 had Down Syndrome
9	McKeon *et al*., 2013.	17	Pilot study to test 2 instruments used to measure PA of men with ID prior to use in larger study	IPAQ & Sensewear armband	Sensewear armband worn for 7 days all the time except when washing or swimming	PA	Ireland	Mean age 42 years. 19-39 yrs = 5 40-59= 12	17 men	6 mild, 2 mod, 9 severe & profound
10	Fitz Gerald & Hahn, 2014.	17	Determine if self-report health status influences physical activity	PAM for objective measurement of activity. Reported by interview the exercise and activity inventory reporting of activities, types and frequency per week	Personal activity monitor (PAM)	PA but did capture hours SB/day	USA (LA)	18 to 59yrs. Mean age men= 33.9yrs Mean age women= 35.7 yrs	10 male, 7 female	Not specified
11	Johnson *et al*., 2014.	37	To examine evidence of convergent and discriminant validity for self-report with assistance from secondary source as a measure of PA in adults with ID	NHANES III PA survey and Actiwatch accelerometer and Omron HJ-112 pedometer	NHANES III PA survey and Actiwatch accelerometer and Omron HJ-112 pedometer	PA	US	19-74 years	21 females, 16 males	Not specified. 11 had DS
12	Hsieh *et al*., 2015.	4282	Examine (1) the impact of three adulthood stages– younger (20–39 years), middle (40–59 years), and older (60 years and older) on BMI & PA, (2) the relationship between social-environmental context (i.e., residence type, everyday choices, and community participation) and BMI and PA	Question on a questionnaire	None. Single question on a questionnaire	Inactivity	USA	20+ yrs	56.6% male, 43.4% female	42% mild, 30.1% mod, 15% severe, 12.8% profound
13	Melville *et al*., 2015	102	Examine effectiveness of walking intervention to reduce SB and increase PA.	Accelerometer	Actigraph GT3X. Min data was 3 days from 7, with minimum 6 hours data on it	PA	Scotland	Mean age in walk well group= 44.9yrs (SD 13.5). In control = 47.7yrs (SD 12.3)	45 females, 57 male	58 mild, 35 moderate, 8 severe
14	Carlson, 2016.	17	To examine if there is a relationship between PA and physical functioning in adults with DS	PA levels assessed by wear of accelerometer for 7 days. Compared to the results of physical functioning tests. Sedentary behaviour classified as any movement <100 hz. Daily sedentary value determined by subtracting the sleep time of each participant. Time spent watching TV (called media time) was recorded with the health history questionnaire.	Triaxial accelerometer (GT3X+, Actigraph, Pensacola, FL)	Both	USA	Mean age 33 years +/-15yrs	8 women, 9 men	Down syndrome
15	Hsieh *et al*., 2017	1618	Investigated the prevalence of reported low levels of PA and hours spent watching TV	Mixed methods, Mail and online survey for data collection	Survey	SB (TV viewing)	US	Mean age 37.67 yrs (18 to 86 yrs)	893 men, 725 women	52.4% mild/mod, 12.4% borderline 8.2% severe or profound
16	Oviedo *et al*., 2017.	84	Sedentary behaviour and physical activity	Accelerometer data over 4 days/week with min of 10hr/day wear	Actigraph GT3X		Spain Catalonia	44 +/-12 years	49 male, 35 female	30 mild, 34 mod, 28 severe
17	Chow *et al*., 2018.	67	Describes the habitual daily physical activity (PA) and the health-related physical fitness (PF) of adults with mild and moderate ID.Secondary focus is determine health-related PF components explain the variance in PA levels and SB	Device worn all waking hours except for bathing and bedtime. Wore for at least 5 consecutive weekdays. Not worn on weekends. Used Freedson to categorise activity types, MVPA>1951 counts/min, Sedentary <100 counts/ min and light intensity was classed as in between	WGT3X-BT Activity Monitor; Actigraph LLC	PA	Hong Kong	Mean 41.7 yrs. 18-64 yrs	71 males, 43 females	Mild & mod ID
18	Melville *et al*, 2018.	725	SB prevalence and correlates	Demographic and health data collected during a structured interview and physical examination. Screen time is measure of SB	Question ' on average how many hours do you spend watching TV, DVDs, videos or on a PC? Response used 9point scale: none, 1-3hrs/ month, 1 hr/ week,2-4hrs/ week, 5-6hrs/ week, 1hr/day, 2-3hrs/ day, 4-5hrs/ day or 6+hrs/ day	SB (screen time)	Scotland, UK	Mean age = 43.6 yrs (18-90 yrs)	55% men (399), 45% women (326)	258 mild, 192 mod, 130 severe, 145 profound
19	Moss & Czyz, 2018.	58	Determine level of agreement between objectively measured Actiheart and IPAQ	Actiheart monitors for 7 days, only removing for bathing. Caregivers completed IPAQ-S	Actiheart activity monitor. Measures and calculates activity energy expenditure based on accelerometry and heart rate measurements	Both	South Africa (NW province)	39.6 years +/-9.1	28 female & 28 male	Mod to mild
20	Woods *et al*., 2018.	19	Determine associations between body composition, diet, PA and timed walk for adults with PWS	Accelerometer worn for upto 7 days. Goal was min of 4 days.	ActivPal accelerometer.	PA	USA Oklahoma	18-62 years	11 male, 8 female	Not specified
21	Harris *et al*., 2019.	143	Investigate the patterns of objectively measured sedentary behaviour in adults with ID	Sedentary behaviour variables output from accelerometer	actiGraph GT3X + accelerometer	SB	Scotland	54 < 45yrs, 86 > 45yrs	69 male, 74 female	69 mild, 51 moderate, 18 severe, 4 profound
22	Oviedo *et al*., 2019.	97. 37 from active group, 29 non- active, 31 no ID	Objective investigation of PA levels and sedentary behaviour in groups of adults with ID and without	Accelerometer. Needed to be worn for >10hrs/day for 4 days/week	Actigraph GT3X	Both	Spain	43+/-12 years (20-60 years)	51 male, 41 female	18 mild to 48 mod
23	Tyrer *et al*., 2019	920	Determine prevalence of multimorbidity in adults with ID and identify risk factors	Cross-sectional analysis	Data analysis	Both	UK	Mean age 42.9 yrs (18-74yrs)	530 male, 390 female	259 mild, 243 mod, 310 severe/ profound
24	Bellicha *et al*., 2020.	10	To objectively quantify spontaneous PA in adult patients with Prader-willi syndrome	Habitual PA. wear an accelerometer for 7 consecutive days during waking hours but not water based activity (3 valid days min with wear time of 8 hours per day). Freedson cutoffs used for sedentary behaviour	Tri‐axial GT3x Actigraph accelerometer	PA	France	18-60 years	10 females	Not specified
25	Ghosh, 2020.	52	To examine levels and patterns of SB in adults with ID	Accelerometer data. Attached to waist during waking hours. Valid accelerometer data was >= 10 hours/ day for 4 days including at least 3 weekdays & 1 weekend day	WGT3X-BT accelerometer (ActiGraph, Pensacola, FL) and data obtained using ActiLife 6 Software v.6.13.4.	SB. SB time as time spent below a threshold of < 100 cpm	US	20-79 years. Median 48	25 men, 27 women	4 men & 6 women had DS, 1 cerebral palsy. 5.8% severe ID. Rest mild to mod

The reasons for study exclusion are shown in Figure 1.

### 3.3 Data extraction

An excel spreadsheet served as the data extraction tool which captured 11 different categories from each study. This was used to summarise all the shortlisted studies. The categories that were captured are shown in a table which is available as extended data ( Lynch, 2021).

***3.3.1. Data items*.** The PICO framework was used to define what data will be sought from variables as follows:

● P: Adults with an Intellectual Disability
  ○ Age, gender, living circumstance, country, number in study, level of ID

● I: Sedentary behaviour
  ○ Level, types of behaviour, quantify change

● C: Level of sedentary behaviour or physical inactivity
  ○ Level, intensity, types of activity/sedentary behaviour, type of employment

● O: Prevalence of sedentary behaviour

***3.3.2. Outcomes and prioritisation*.** The outcome of this investigation into sedentary behaviour determined the sedentary behaviour levels of older adults with an intellectual disability.

***Primary outcome***

- Sedentary behaviour levels

### 3.4. Data Synthesis

Article data was grouped according to the sedentary behaviour (SB) assessment category used in each article. Four methods for quantifying SB were identified in the 25 articles that passed the quality assessment. These four methods were:

1. Number of steps per day
2. Amount of screen time per day
3. Time in sedentary behaviour (SB) per day
4. Different methods

The data was scrutinised to establish the breakdown of SB time, steps and screen time by residence, age, level of ID and gender but this was not always possible because studies often used different age ranges e.g. 18–49, <45, 50+, and few studies analysed results by residence type, gender or ID level. Thus, this type of analysis was not always possible.

RevMan Review Manager Version 5.4.1 ( The Cochrane Collaboration, 2020) was used to synthesise results in a graphical format called a Forest Plot. A Forest Plot is a graphical representation of a meta-analysis where individual study’s results are represented by a box, and lines which denote the 95% confidence interval (CI). The influence a study has on the overall meta-analysis, the study weighting, is denoted by the size of the box. The amount of result variance between individual studies is represented by the heterogeneity value, I ^2^, of the forest plot ( Israel & Richter, 2011). A higher I ^2^ value means a greater difference is observed between studies which is not due to chance and a meta-analysis may be inappropriate, as studies may not have similar populations. Values for I ^2^ of greater than 50% are considered to be indicative of moderate heterogeneity, 75% or greater is considered high, while values of 25% are low and hence similar ( Higgins *et al.*, 2003). A random effects model was used in the Forest plots to account for variance in studies such as varied settings, measurement devices, age or mixed levels of ID.

The mean difference and standard error for each study were used to determine a pooled mean prevalence for SB. In addition, a cumulative mean of means was calculated to determine the pooled prevalence of SB. Pair-wise comparisons were calculated where data was available using means and standard deviations. Scales were adjusted on the Forest plots so results may be seen clearly.

## 4.0 Results

### 4.1. Measurement devices

A variety of measurement devices were used to assess sedentary behaviour. The prominent devices for measurement were accelerometers which were used in 14 studies. However, 4 used pedometers, 1 used a personal activity monitor, 1 used a survey as well as pedometers and accelerometers, 3 used a questionnaire or survey, 3 used IPAQ and accelerometers and 2 studies used self-report and accelerometers.

### 4.2. Steps per day

Steps as a measure of physical activity or SB were used in 11 studies, which involved 985 participants. The objective measurement of steps per day in these 11 studies was obtained using accelerometers and pedometers as shown in Table 4, which also shows the mean and range of steps per day. As can be seen in Table 4 a variety of devices were used.

**Table 4.** Studies that used steps to determine SB/PA.

Article no	Article name	Measurement device	No of participants	Steps per Day Mean (SD)	Step Range [Low -high]
1	Temple & Walkley, 2003	Accelerometer [Caltrac]	37	8100 (3735.4)	1,658 - 19,303
2	Peterson *et al.,* 2008	Pedometer [Omron Hj-700IT]	131	6,506 (3296)	1,703 - 24,369
3	Finlayson *et al.,* 2011	Accelerometer [ActivPal], Self-report	62	8509 (4384)	380 - 21,139
4	Hilgenkamp *et al.,* 2012	Pedometer [NL1000]	257	6601 (3610)	NA
5	Bergström *et al.,* 2013 Bergström *et al.,* 2013	Pedometer [LS2000]	130	8,042 (5,524) [Int] * 6,296 (4167) [Ctrl] *	NA
6	Dixon-Ibarra *et al.,* 2013	Accelerometer [GT1M Actigraph], pedometer [Omron HJ720ITC]	109	Done by age	NA
7	Mckeon *et al.,* 2013	Accelerometer [Sensewear armband], IPAQ	17	5,308 (5,502)	44 - 21,219
8	Johnson *et al.,* 2014	Accelerometer [Actiwatch], pedometer Omron [HJ112], survey [NHANES III]	37	6,625.4 (3,303.72)	NA
9	Melville *et al.,* 2015	Accelerometer [Actigraph GT3X]	102	4,780 (2432)	NA
10	Oviedo *et al.,* 2017	Accelerometer [Actigraph GT3X]	84	6,192 (2814)	NA
11	Woods *et al.,* 2018	Accelerometer [ActivPal]	19	7,631.7 (1171)	NA

*=Pre-intervention, NA=Not available

An RCT by Melville and colleagues observed that at baseline the 102 Scottish participants, who had mild to severe level of ID and lived in different residential settings, were sedentary for 65.5% of the day ( Melville *et al.*, 2015). Being female, older age, more severe ID and having mobility impairments were significant predictors for low levels of PA ( Hilgenkamp *et al.*, 2012).

***4.2.1 Steps per day and age*.** Some studies found that age could be a contributing factor to the number of steps per day taken. A US based cross-sectional study investigating the sedentary behaviour of two different age groups of adults with ID, younger adults (aged 18–49 years) and older adults aged 50+, showed the average steps per weekday decreased with age. However, the authors felt this difference could be attributed to the younger group having more wear time. More than 40% of adults with ID and more than 55% of older adults with ID had <5000 steps per day ( Dixon-Ibarra *et al.*, 2013). Similarly, in the Dutch based Healthy Aging and Intellectual Disability (HA-ID) study, 257 eligible older adults aged 50+ years of all levels of ID and residential settings wore a pedometer for 14 days. The average number of steps per day and the number in each age group that met the daily step recommendation was inversely proportional to age groups. In the 50–59 years group (n=146) 17.8% had greater than 10,000 steps per day and 41.1% had greater than 7,500 steps per day. In the 60–69 years (n=83) 18.1% >10,000 and 34.9% >7,500 steps per day. In the 70–79 years group (n=25), 8% > 10,000 and 16%>7,500. In the 80–89 years group (n=3) no one had greater than 7,500 steps per day. Overall, 39% of participants performed <5,000 steps per day ( Hilgenkamp *et al.*, 2012).

Conversely, Woods *et al.* (2018) which examined the behaviour of 19 participants aged 18 to 62 years with Prader-Willi Syndrome, found the 18–30 years and 40+ age group had similar steps but the 30–40 years had less steps. A study with 131 US-based ambulatory community living adults with ID showed that ID and age were strong factors in the numbers of steps per day taken ( Peterson *et al.*, 2008). Conversely a Spanish study with 84 adults who had varying levels of ID and attended an occupational day centre observed no difference in age-related SB ( Oviedo *et al.*, 2017). Hence the age and step count per day relationship is inconclusive. A summary of studies with age-related steps per day is shown in Table 5.

**Table 5.** Steps per day by age group.

Article no	Article Name	Age (years)	Steps per Day Mean (SD)
1	Dixon-Ibarra *et al.*, 2013	18–49	6831 (±3221)
	Dixon-Ibarra *et al.*, 2013	50+	4596 (3052)
2	Hilgenkamp *et al.*, 2012	50–59	7038 (3565)
	Hilgenkamp *et al.*, 2012	60–69	6578 (3699)
	Hilgenkamp *et al.*, 2012	70–79	4616 (2818)
	Hilgenkamp *et al.*, 2012	80–89	2511 (1336)
3	Woods *et al.*, 2018	18–30	8243.19 (2237.1)
	Woods *et al.*, 2018	30–40	5411.51 (1379.84)
	Woods *et al.*, 2018	40+	8379.74 (1660.86)

***4.2.2 Steps per day and gender*.** Some studies found that gender was a contributing factor to less steps per day. This was investigated by four studies. A 2011 Scottish study with 62 community-based adults with mild to moderate ID deduced that women were significantly more likely to be sedentary ( Finlayson *et al.,* 2011). However, Johnson and colleagues in a study investigating physical activity levels of 37 community-based ambulatory adults with ID found the average daily step count accumulated over 14 days was comparable for both genders ( Johnson *et al.*, 2014). Similarly, a study with 19 participants with Prader-Willi Syndrome found the mean steps per day for males was analogous to females ( Woods *et al.*, 2018). In contrast, the Dutch HA-ID study, found that 21.8% of male participants and 11.3% of females had >=10,000 steps/day, while 42.9% men and 29% women had >=7500 steps/day ( Hilgenkamp *et al.*, 2012). Hence the effect of gender on steps per day is inconclusive. Table 6 shows the mean steps per day by gender.

**Table 6.** Mean Steps per day by gender.

Article no	Article Name	Female Steps/day Mean (SD)	Male Steps/day Mean (SD)
1	Finlayson *et al.*, 2011	6481 (2998)	11,101 (+/-4575)
2	Hilgenkamp *et al.*, 2012	5966 (2937)	7193 (4063)
3	Johnson *et al.*, 2014	6809.63 (3056.2)	6406.72 (3693.61)
4	Woods *et al.*, 2018	7894.3 (2021.1)	7325.4 (1173.6)

**Figure 2.**
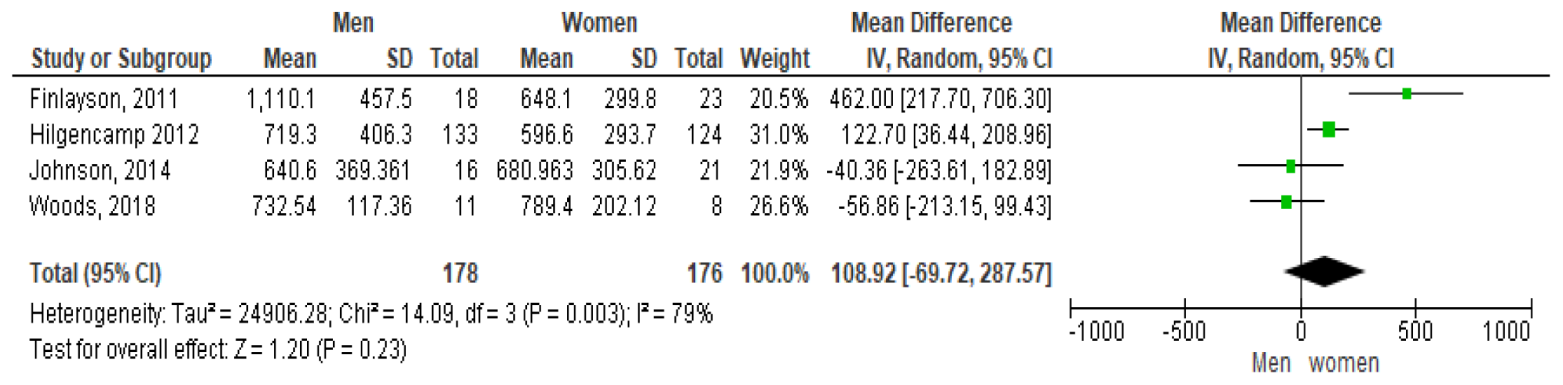
Pairwise comparison of steps per day by gender (divided by 10).

The forest plot shown in Figure 2 shows the gender pairwise comparison. According to this plot females take more steps per day than males, which is contrary to some study results ( Westrop *et al.*, 2019). The mean difference seen is 1,089.2 steps per day at 95% CI [-69.72, 287.57]. However, a high heterogeneity of I ^2^ = 79% is observed indicating it may not be appropriate to pool article results due to study differences ( Higgins *et al.*, 2003). In addition, as the diamond shape touches the line of no effect the overall effect is not significant.

***4.2.3 Steps per day and day of week*.** Several studies highlighted the influence of weekday versus weekend on the daily step count. The Dixon-Ibarra *et al.* (2013) study showed significantly less steps were observed from weekdays to weekends for all adults with ID. For weekends, adults with ID had an average of 4530 (SD±2337) steps per day and older adults with ID had 3504 (SD±2239). Finlayson and colleagues (2011) also found participants were more active on weekdays than weekends. Similarly, the average step levels in a Spanish study ( Oviedo *et al.*, 2017) were higher on weekdays with 6523 (SD±2807) steps per day compared to 5378 (SD±3686) steps per day at the weekend Equally, Peterson *et al.* (2008) found that weekday steps per day ranged from 1796 to 21,744 while weekend steps per day ranged from 1189 to 30,931. There appears to be an influence of weekend versus weekday on step levels.

**Figure 3.**
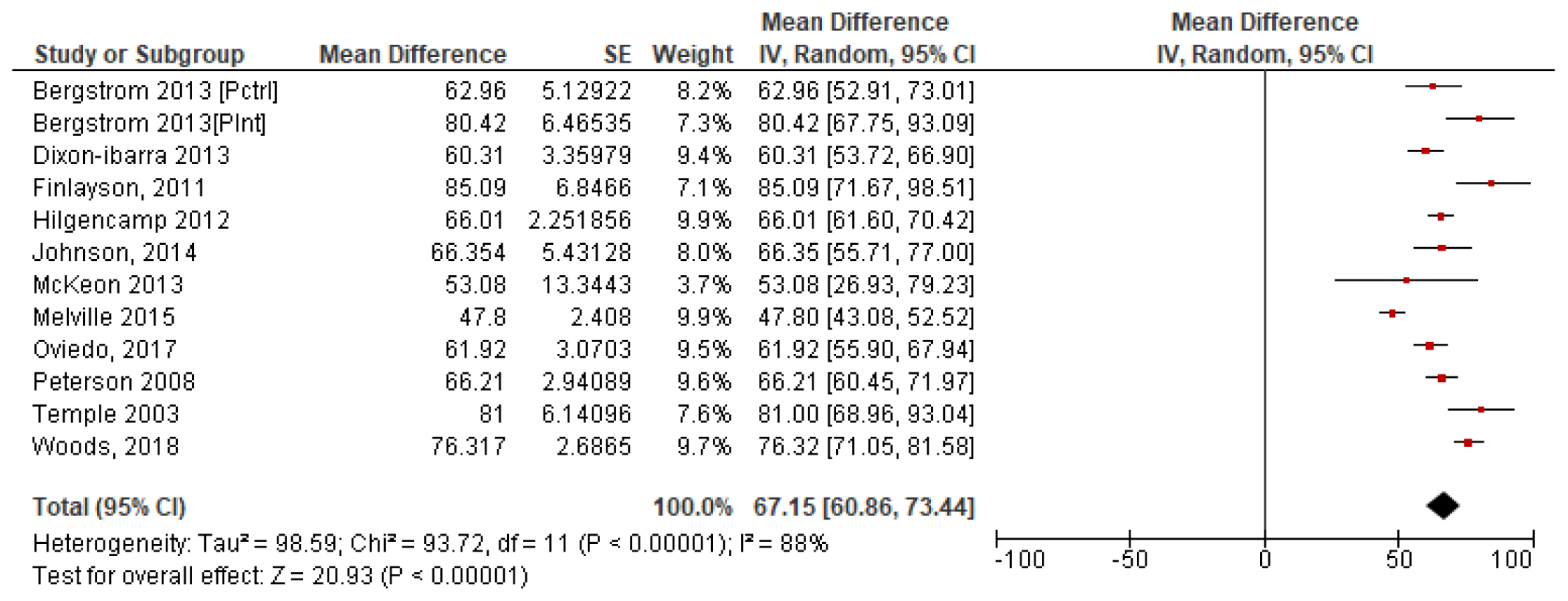
Study steps per day means with SE ( *Divided by 100*).

***4.2.4 Summary steps per day*.** To calculate a pooled mean of steps per day, a forest plot was produced using each of the 11 study’s individual mean and standard error. The results which give a pooled study mean of 6,715 steps per day, at 95% confidence interval (CI) [6,086, 7,344] are shown in Figure 3. The variability between studies is very high with I ^2 ^=88% indicating high heterogeneity, which may indicate that it is inappropriate to combine studies due to the potential variability in studys ( Higgins *et al.*, 2003).

### 4.3. A cumulative mean of means was calculated for all 11 studies. This pooled mean result was 6,555 steps per day. Screen time

**Figure 4.**
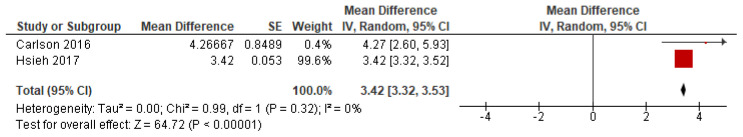
Screen time.

In total three articles used television (TV) viewing as a means of evaluating SB. Two articles quantified SB by the amount of time spent looking at a screen, whether that was watching TV, videos, DVDs, using a gaming console or computer. The third article, Melville *et al.* (2018) in a cross-sectional study of 725 people with an ID, used a proxy-based measure of subjective screen times. This showed that 50.9% of participants spent four or more hours per day watching TV. This study showed that increased screen time was associated with higher levels of ID, being male, having mobility issues, obesity, hearing issues and epilepsy. The second study, with 1,618 participants, which was a mixed methods study using mail and an online survey to gather information indicated that 61.5% of participants watched three or more hours of TV per day and 40% watched four or more hours per day ( Hsieh *et al.*, 2017) which was a similar time observed in the Carlson study which had 17 participants ( Carlson, 2016). Hsieh and colleagues (2017) also found that men with ID spent more time watching TV than women with ID. Furthermore, time spent watching TV was higher for those living on their own or in family homes than group homes. Those with mild/moderate ID spent more time watching TV than severe/profound. No difference in TV watching was observed by age groups. Figure 4 illustrates the forest plot for screen time. This plot has low heterogeneity with I ^2^=0%, which indicates that the two studies may be compared. It shows the mean screen time per day is 3.42 hours at 95% CI [3.32, 3.53].

### 4.3. Assessing sedentary behaviour by time

SB, which is time spent sedentary, was assessed using time (in either hours or minutes per day) in 13 studies which included 713 participants. Objective measurements were obtained by accelerometers and/or pedometers and in one case a personal activity monitor. The minimum sedentary activity observed was 4hrs/day and the maximum 24hrs/day ( McKeon *et al.,* 2013). A study with 17 participants with ID, showed that higher ratings of self-reported health status predicted less SB and greater PA minutes in persons with ID ( Fitz Gerald & Hahn, 2014). A larger sample in a Spanish study which compared the activity and SB of 66 active and non-active individuals with mild and moderate ID and 31 older adults with no ID, found there were large amounts of SB even if groups met the PA guidelines for health. Furthermore, the number of sedentary bouts was greater in the ID groups than non-ID groups ( Oviedo *et al.*, 2019). Sedentary time was accumulated in bouts of 1–30 minutes in duration in a US-based study with 52 participants with all ID levels ( Ghosh, 2020). Harris and colleagues (2019) demonstrated that 143 participants had a median of 7 breaks per day (95% CI, 4-11), where the median duration of breaks observed was 43.2 minutes (95% CI, 27.2-73.7). An accelerometer and the Bouchard scale were used to quantify activity levels of 37 participants with mild to moderate ID in Australia. The nine-point Bouchard scale defined level 1 as lying down, sleeping or resting and level 2 as seated activity. Using level 1 and 2 as indicators of SB, participants were sedentary for an average of 83.7% of each day ( Temple & Walkley, 2003). Another Spanish study found that 84 adults with varying levels of ID who attended an occupational day centre spent 79.4% of their waking hours sedentary ( Oviedo *et al.*, 2017). A study looking at activity levels of 90 adults with ID living in group homes identified that participants are extremely sedentary during weekdays, spending the largest percentage of time in SB (mean = 67.3%, SD±12.0%) ( Chow *et al.*, 2018). These studies and the mean sedentary time per day are shown in Table 7.

**Table 7.** Studies that used time to assess sedentary behaviour.

No	Article name	Measurement device	No of participants	SB per day (Hours)
1	Temple & Walkley, 2003	Accelerometer [Caltrac]	37	20.105 (4.73)
2	Finlayson *et al.*, 2011	Accelerometer [Activpal], Self-report	62	18.71 (1.88)
3	Dixon-lbarra *et al.*, 2013	Accelerometer [Actigraph GT1M], pedometer [Omron HJ720ITC]	109	NA
	Dixon-lbarra *et al.*, 2013 [18–49Yrs]	Actigraph GT1M & Omron HJ720ITC	40	6.75 (1.94)
	Dixon-lbarra *et al.*, 2013 [50+Yrs]	Actigraph GT1M & Omron HJ720ITC	28	7.35 (1.77)
4	McKeon *et al.*, 2013	Accelerometer [Sensewear armband], IPAQ	17	15 (6)
5	Fitz Gerald & Hahn, 2014	Personal activity monitor, interview	17	NA
	Fitz Gerald & Hahn, 2014 [Males]	Personal activity monitor, interview	12	22.9 (0.47)
	Fitz Gerald & Hahn, 2014 [Females]	Personal activity monitor, interview	10	23.2 (0.19)
6	Carlson, 2016	Accelerometer [Actigraph GT3X]	17	7.28 (1.33)
7	Matthews *et al.*, 2016	Accelerometer [Actigraph GT1M]	45	10.17 (2.06)
8	Oviedo *et al.*, 2017	Accelerometer [Actigraph GT3X]	84	10.22 (1.34)
9	Chow *et al.*, 2018	Accelerometer [Actigraph WGT3X-BT]	90	8.25 (1.45)
10	Harris *et al.*, 2019	Accelerometer [Actigraph GT3X]	143	8.1 (2.1)
11	Oviedo *et al.*, 2019	Accelerometer [Actigraph GT3X]	66	NA
	Oviedo *et al.*, 2019 [Active group]	Accelerometer [Actigraph GT3X]	37	10.25 (1.78)
	Oviedo *et al.*, 2019 [Nonactive group]	Accelerometer [Actigraph GT3X]	29	10.25 (1.34)
12	Bellicha *et al.*, 2020	Accelerometer [Actigraph GT3X]	10	8.712 (0.363)
13	Ghosh, 2020	Accelerometer [Actigraph WGT3X-BT]	52	8.6

With consideration to differences observed in SB between weekends versus midweek, three studies did identify differences but not consistently. Table 8 shows the measured SB in three such studies. Furthermore, a secondary analysis of two pooled RCTs showed a significant difference in break duration between weekdays 79.8 (SD ±151.6) minutes and weekend days 62.6 (SD ±55.7) minutes ( Harris *et al.*, 2019).

**Table 8.** Weekend versus weekday sedentary behaviour.

No	Article name	SB Weekday (Hours)	SB Weekend (Hours)
1	Oviedo *et al.*, 2017	10.4 (1.39)	9.73 (1.7)
2	Finlayson *et al.*, 2011	18.49	19.28
3	Harris *et al.*, 2019	8.2	8

**Figure 5.**
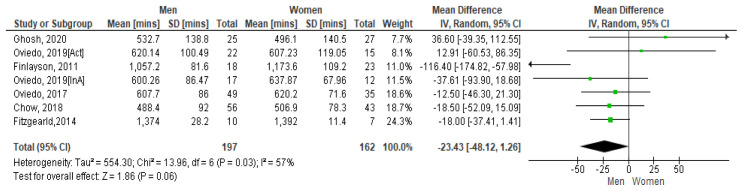
Mean sedentary minutes by gender (divided by 10).

***4.3.1 Sedentary time and gender*.** A 2011 Scottish study with 62 community-based adults with mild to moderate ID presented the average SB time per day for women as 19.56 hours and men 17.62 hours. On weekdays this was 19.46 hours for women and 17.24 hours for men ( Finlayson *et al.*, 2011). Figure 5 shows the paired comparison of mean sedentary minutes per day by gender. The Spanish study which had results for active (Act) and inactive (InA) groups of participants show both groups included separately here. This forest plot shows that men have less sedentary minutes per day than women, the mean difference is -234.3 [95% CI, -48.12, 1.26]. The Fitzgerald study appears to have the biggest influence on the pooled result due to its higher weighting. However moderate heterogeneity is present between studies as demonstrated by I ^2^=57%. Hence it may be inappropriate to combine results ( Higgins *et al.*, 2003).

**Figure 5.1.**
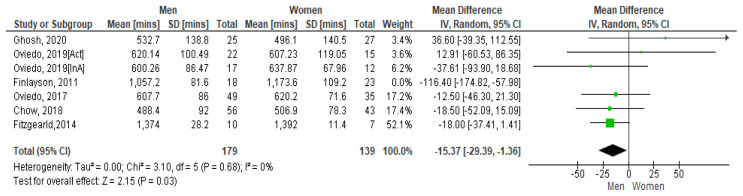
Mean sedentary mins by gender (excluding Finlayson) (divided by 10).

However, if the Finlayson study is excluded from the calculation (as it appears to be an outlier), the I ^2^ value reduces to zero. See Figure 5.1. The Forest plot still shows that men have more sedentary minutes than women and as the lower and upper points of the horizontal plane of the diamond (i.e. the [95% CI, -293.9, -1.36]) both lie to the left, it means the resulting difference is significant. The mean difference is -153.7. No heterogeneity is present so it is appropriate to combine study results.

***4.3.2 Summary SB time*.** The percentage of waking time spent in SB seen in these studies varied from 72% of wear time ( Bellicha *et al.*, 2020) to 83.77% ( Temple & Walkley, 2003). The total daily time observed in SB in these studies varied from 437minutes or 7.28 hours to 1206.3 minutes or 20.1 hours.

**Figure 6.**
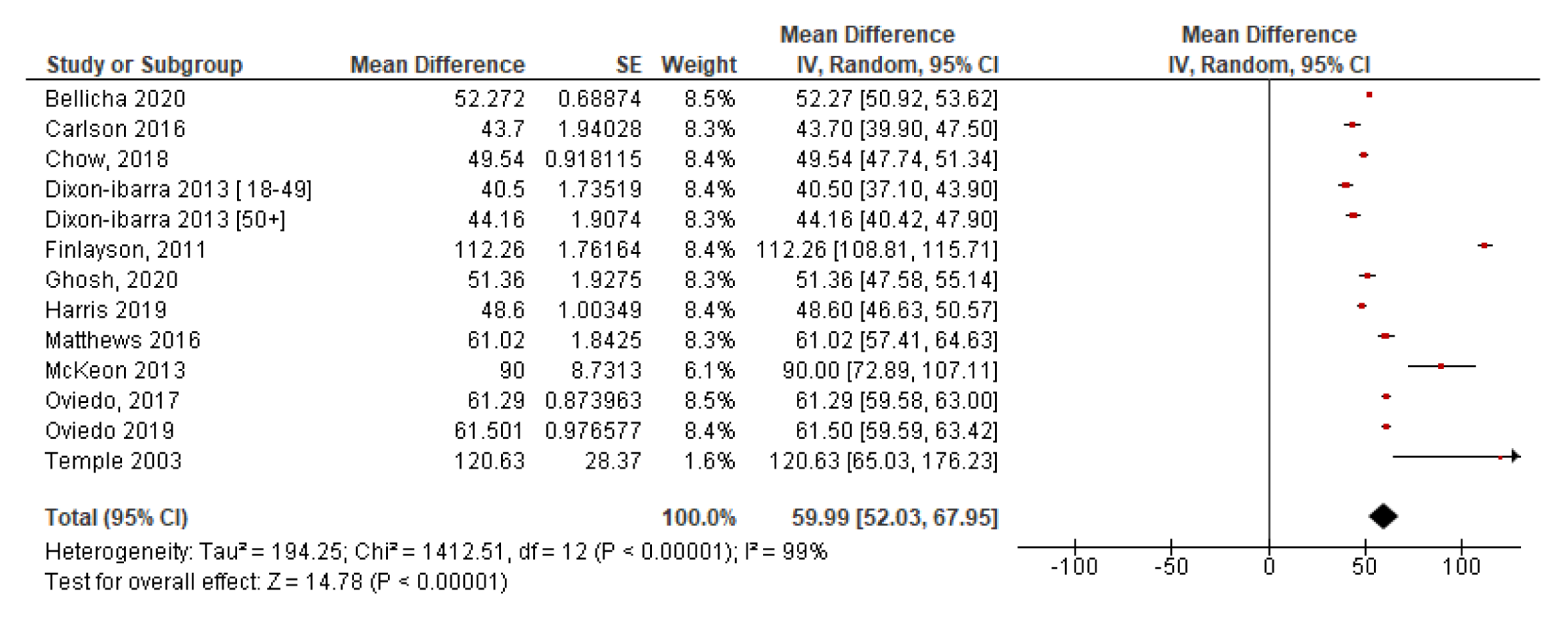
Mean time sedentary with SE ( *minutes divided by 10*).

For analysis purposes, all times were converted to minutes. Figure 6 shows a forest plot of studies with mean sedentary time in minutes. This demonstrates high heterogeneity (I ^2^=99%) and hence high levels of variability among study results which means it may be inappropriate to pool results ( Higgins *et al.*, 2003). However, the plot provides a good visual representation of the results. The mean sedentary minutes was 599.9 minutes per day at 95% CI [520.3, 679.5] or 9.99hours. A pooled mean result for all studies was calculated using a mean of means formula (as used in Section 4.2). The resulting pooled mean of total sedentary minutes per day for all 13 studies is 606.3minutes or 10.1hours per day.

### 4.4. Diverse methods

Two studies used alternate methods to identify SB. The first study with 58 participants with ID using an ActiHeart device investigated Physical activity level (PAL). PAL is the ratio of total energy expenditure and resting energy expenditure as described by United Nations Food and Agriculture Organisation (2001). PAL cut-off points for activity levels were <1.4 for sedentary. The mean total physical activity level measured in this study was 1.39 (SD+/-0.15) which is indicative of a sedentary lifestyle ( Moss & Czyz, 2018). The second study which used a question on how much sitting time people did to identify SB, found that 47% of the 920 participants sat for ‘all, most or a lot of the day’ ( Tyrer *et al.,* 2019).

## 5.0. Discussion

Sedentary behaviour is associated with poorer health and earlier mortality ( Patel *et al.*, 2010). However, specific guidelines for sedentary levels do not exist for the general population or people with intellectual disability. The WHO recommend minimising the amount of time in sedentary behaviour and replacing it with physical activity of any type or intensity for health benefits ( WHO, 2020c). This review shows that there are limited studies investigating the SB of people with an ID. In total, the number of participants represented in this review are 9,111. Overall, the results of this study identified that adults with ID were sedentary for over 60% of waking hours and on average the participants took almost 6,500 steps per day. These identified sedentary levels are similar to other studies in the intellectual disability population ( Harris *et al.*, 2019; Melville *et al.*, 2018) and whilst the steps per day did not meet the recommended 10,000 there appears to be some level of activity. That said it must be kept in mind that the sampling in the studies was limited to those with a mild/moderate level of ID and had no mobility difficulties therefore the picture emerging may not represent the entire story as those with a more severe, profound or multiple complex health are not included. These are the very individuals who most need to be active. Along with that, the available studies have taken very different approaches to establishing SB. Hence, it is difficult to derive definitive conclusions from the data presented.

It appears that consistent methods for gathering SB data were not used across studies. A diverse range of measurement devices were used for taking objective measurements. These include Actigraph, Actiwatch, ActivPal, Sensewear armband, Caltrac accelerometers, Omron pedometers and a Personal Activity Monitor. While the Actigraph was the predominant choice for objective measurements in these studies, it has been shown that the Actigraph accelerometer may not be the most accurate for assessing SB due to device placement at the hip and resulting postural measurement limitations ( Aguilar-Farías *et al.*, 2014; Kim *et al.*, 2015). Thus, results in the studies that used an Actigraph may be questionable. Furthermore, two studies which used accelerometers and the IPAQ questionnaire to assess SB determined there was low level of agreement between the two methodologies, with the IPAQ significantly underestimating sedentary time ( Matthews *et al.*, 2011; Moss & Czyz, 2018). In addition, there were three studies that used either self-reported methods, interviews or surveys to garner the SB information. In summary the measurement of SB in adults with ID is inconsistent across studies. The methods used are not always comparable and results may not be reliable. However, steps was seen to be commonly reported across most of the studies.

Steps were used as one of the assessment types for determining SB in 11 studies. The general consensus is that taking 10,000 steps per day is necessary for health ( Wattanapisit & Thanamee, 2017). Dixon-Ibarra *et al.* (2013) showed that 10% (n=4) of 18–49-year-old adults with ID and 3% (n=1) of 50+-year-old adults with ID achieved the recommended 10,000 steps per day however the sample size who attained this level of activity is small so not generalisable and does conjure up the question if this level of steps is attainable for all those with ID. Another study showed that 3 people (15%) had >= 10,000 steps/day, while several had <5000 steps per day, which according to some experts is indicative of a sedentary lifestyle. However, these findings must be viewed with caution considering the sample size they are based on was only 19 ( Tudor-Locke *et al.*, 2013; Woods *et al.*, 2018). Similarly, Finlayson and colleagues (2011) presented that 27% of 62 participants achieved 10,000 steps per day which is higher than seen in other studies In summary these studies show that few people with an ID are achieving the recommended 10,000 steps per day for health. This compares to an average of 9,448 steps per day in the general population, which reduced to 6,565 in those aged over 65 years as determined by a meta-analysis ( Bohannon, 2007).

In contrast to steps the WHO recommends minutes/week of physical activity to promote health benefit for all adult populations. They note that adults should achieve a minimum of 150 minutes of moderate to vigorous physical activity (MVPA) per week ( WHO, 2019). Tudor-Locke and colleagues (2011) pronounce that this translates to 7,100 steps/day. Similarly, Cao *et al.* (2014) recommend the step equivalent for meeting minimum recommended activity levels to be 7,700 per day. The meta-analysis in this study pooled mean steps per day were calculated between 6,430 and 6,555 respectively falling short of the WHO recommendations. Unfortunately, this highlights the fact that the average steps per day levels of people with ID do not meet adequate levels to achieve minimum activity recommendations and hence the associated health benefits. This is an overall concerning finding considering the implications to overall health, including increased metabolic risks, diabetes and all-cause mortality ( Biswas *et al.*, 2015; Edwardson *et al.*, 2012; Krishnan *et al.*, 2009). This is concerning considering that almost 80% of participants in the IDS-TILDA study were identified as being either overweight or obese and over 70% did not meet the required activity levels ( Burke *et al.*, 2017; McCarron *et al.*, 2017). Additionally, multimorbidity rates have been identified between 71–98% ( Kinnear *et al.*, 2018; McCarron *et al.*, 2013). Meeting the minimum recommended activity levels has been shown to increase perceived health status as well as quality of life indicators ( Brown *et al.*, 2003).

Only four studies looked at the relationship between steps and gender. The pooled results indicated that women took more steps per day than men which was possibly due to the influence of the weighting of the Hilgencamp study on the mean which had the largest number of participants (of both genders) with 257 ( Hilgenkamp *et al.*, 2012). This pooled analyses appear to have a high inter-study variability as demonstrated by the I ^2^ value of 79% so results may not be definitive In contrast, a pooled analysis which looked at the gender influence on the mean sedentary minutes per day showed men having less sedentary minutes than women per day. This analysis appeared to have equivalent weightings for all 6 studies. Westrop’s systematic review investigating gender differences in SB observed no statistical differences for SB by gender, but women with ID were found to be less active than men ( Westrop *et al.,* 2019). This is important as generally the research points to women with ID being at greater health risk for example of morbid obesity and diabetes ( Burke *et al.*, 2017; Hsieh *et al.*, 2014; Hsieh *et al.*, 2015).

Another assessment type used to determine SB levels was time. Considering there are 1,440 minutes per day and if nine hours (=540 minutes) are spent sleeping, this means there are 900 minutes per day available for activity ( Carlson, 2016). The pooled mean time calculated from the 13 studies that used time to quantify SB was 556.5 minutes or 9.3hours, 61.8%, per day which is equivalent to the calculation done using a mean of means formula which resulted in 606.31 minutes or 10.1hours or 67.4%, time in SB per day. This is a huge amount of time to be sedentary every day and the potential health implications for depression, cognitive function, functional ability and quality of life are evident ( Saunders *et al.*, 2020). In the general population sedentary time of 8 to 9 hours a day has been identified in a multi-country analysis, with this time increasing as people aged ( Loyen *et al.*, 2017). Sitting is sometimes recognised as the new smoking, detrimental to health and associated with all-cause mortality ( Chau *et al.*, 2013). Considering that the majority of people with intellectual disability may have underlying health issues, this level of SB can only be devastating to their health. Comparable SB levels of 65.5% were observed by Melville *et al.* (2015) who similarly reported the possible catastrophic outcomes should this level continue. Unfortunately, in a more recent study higher levels of SB of 72% and 79.4% have been observed ( Bellicha *et al.,* 2020; Oviedo *et al.*, 2017). Thus, the evidence suggests that sedentary levels of more than 60% a day are normal and prevalent for adults with ID which is very concerning due to the potential health repercussions. Furthermore, sleep time of 9 hours is approximate and may be under or over-representative of the amount of time spent sleeping.

While only four studies provided an analysis of an age influence on SB, those that did had inconsistent results. Some studies identified that age had no influence on SB levels ( Oviedo *et al.*, 2017; Woods *et al.*, 2018), while Hilgenkamp & colleagues (2012) found older age was a significant predictor of low levels of PA but not necessarily SB which was confirmed by Dixon-Ibarra & colleagues (2013) who found that older adults with ID, (50+ years) were found to take significantly less steps than younger adults with ID ( Dixon-Ibarra *et al.*, 2013). While ageing is a time when people tend to slow down ( Donoghue *et al.*, 2016), there is a need to promote active ageing to maintain health as long as possible. Many countries promote positive ageing policies with the philosophy of self-determination ( DoH, 2015), however individuals with ID need more support to attain positive ageing. Ultimate responsibility to provide this support is with support workers and families. It is evident from this systematic review that adults with ID have a highly sedentary lifestyle and the possible negative impact to their health will be great. Conversely studies that investigated a weekday versus weekend influence, appear to see a consistent increase in SB at the weekends compared to weekdays ( Dixon-Ibarra *et al.*, 2013; Finlayson *et al.*, 2011; Oviedo *et al.*, 2017). This warrants further investigation and invites more questions for example about the influence of residence type on weekend activity and overall support for positive ageing.

Screen time was another point of measurement observed in the literature. This analysis demonstrated that the observed pooled average screen time was 3.42 hours per day. A study in the general population using television viewing and work sitting as measures of sitting behaviours found that sitting for more than three hours a day, especially watching television had detrimental effects, specifically for CVD and diabetes ( Pereira *et al.,* 2012). Furthermore, a direct relationship has been observed between adverse health outcomes and TV watching ( Thorp *et al.,* 2011). The IDS-TILDA study showed that less than 20% of the participants regularly used a computer which would imply that the predominant screen time for this population is TV watching ( McCarron *et al.*, 2017). This level and type of screen time ultimately promotes SB which could lead to a degradation in health for people with ID who are already adversely affected by poorer health and higher levels of multimorbidity, diabetes and obesity ( Gawlik *et al.*, 2018; McCarron *et al.*, 2013; Tyrer *et al.*, 2019).

Participants with a more severe or profound ID were excluded from 60% of studies which means a large proportion of individuals with ID were not included in these SB figures. This is very concerning, not only from the perspective of the missing voice of those with this level of ID from the research, but also this is a cohort who are at greater risk of multiple complex health conditions ( Van Timmeren *et al.*, 2016). In fact, McCarron and colleagues found that those with a more severe or profound level of ID were more likely to have more complex health conditions, higher levels of co-morbidity and mobility limitations ( McCarron *et al.*, 2015). If this cohort are excluded from studies this could lead to an underestimation of SB in people with ID. Furthermore, while considering mobility, the inclusion criteria for several studies specified that participants needed to be independently ambulatory ( Chow *et al.,* 2018; Dixon-Ibarra *et al.,* 2013; Fitz Gerald & Hahn, 2014; Harris *et al.,* 2019; Johnson *et al.*, 2014; Oviedo *et al.,* 2017; Oviedo *et al.* 2019; Peterson *et al.*, 2008; Ryan *et al.*, 2014; Temple & Walkley, 2003; Temple, 2007). Participants with severe mobility problems were also excluded ( Bergström *et al.,* 2013; Carlson, 2016; Ghosh, 2020; Hilgenkamp *et al.*, 2012; Melville *et al.,* 2015). Accordingly, the exclusion of less able-bodied individuals by 64% of the studies, results in inaccurate lower observed sedentary levels and does not provide the full picture of SB among those with all levels of ID.

This systematic review confirms that adults with ID are more sedentary than their non-ID peers and high levels of SB are extremely prevalent in people with an ID. It must be noted however that studies were inconsistent in their approach and measurement of SB.

## Conclusion

High levels of time spent in sedentary behaviour are observed in the literature in adults with an intellectual disability, although inconsistencies exist around measurement techniques and tools used to gather data, all papers reviewed confirm these findings. This review has shown that men spend less time being sedentary per day than women, but that women take more steps per day, however studies are very heterogenous. A limitation observed in the studies used for this systematic review is that they do not appear to be fully representative of the ID population as often do not include those with a more severe levels of ID or who have mobility issues. This systematic review and meta-analysis have demonstrated that SB is widespread among the adult population of individuals with ID. There is a need to address this through education and health promotion and further research to establish a full picture of SB is necessary. Additional studies which include objective measurements, adults with all ID levels and mobility levels, and with a primary focus on SB are necessary to accurately determine the prevalence of this type of behaviour.

## Data availability

Harvard dataverse: Replication data for Sedentary behaviour levels in adults with an intellectual disability: a systematic review and meta-analysis." DOI: https://doi.org/10.7910/DVN/HYMA0J. ( Lynch, 2021)

This project contains the following data:

- The extended data included as part of this systematic review are the PRISMA-P checklist, PRISMA-P flow diagram and the excel spreadsheet which contains details on the final 25 articles used in the systematic review.

Data are available under the terms of the Creative Commons Zero "No rights reserved" data waiver (CC0 1.0 Public domain dedication).

### Reporting guidelines

Harvard dataverse. PRISMA checklist and flow chart for ‘Sedentary behaviour levels in adults with an intellectual disability: a systematic review and meta-analysis’. DOI: https://doi.org/10.7910/DVN/HYMA0J

Data are available under the terms of the Creative Commons Zero "No rights reserved" data waiver (CC0 1.0 Public domain dedication).
