# Supplementary material for: Sedentary behaviour levels in adults with an intellectual disability: a systematic review and meta-analysis
Source: HRB Open Res. 2022 Mar 30;4:69. Originally published 2021 Jul 5. [Version 3] doi: 10.12688/hrbopenres.13326.3 (PMC9020532; doi:10.12688/hrbopenres.13326.3)
Supplement: Peer review history

# Peer review: Reviewer response for version 3

**DOI:** 10.21956/hrbopenres.14780.r31764

**Published:** 2022-04-19

**Authors:** Alan Donnelly

**Affiliations:** 1 Department of Physical Education and Sport Sciences, University of Limerick, Limerick, Ireland

All recommended changes have been made, and the paper is now in good order.

Are the rationale for, and objectives of, the Systematic Review clearly stated?

Yes

Is the statistical analysis and its interpretation appropriate?

Yes

Are sufficient details of the methods and analysis provided to allow replication by others?

Yes

Are the conclusions drawn adequately supported by the results presented in the review?

Partly

Reviewer Expertise:

Sedentary behaviour measurement; sedentary behaviour and health.

I confirm that I have read this submission and believe that I have an appropriate level of expertise to confirm that it is of an acceptable scientific standard.

# Peer review: Reviewer response for version 2

**DOI:** 10.21956/hrbopenres.14619.r31241

**Published:** 2022-03-25

**Authors:** Alan Donnelly

**Affiliations:** 1 Department of Physical Education and Sport Sciences, University of Limerick, Limerick, Ireland

Adults with an intellectual disability are often ignored as a population when studying sedentary behaviour, so it is good to see a clearly presented systematic review in the area.

 The review methodology is effective, and reporting of the methods is good.

 I am a little concerned with some laxity in reporting of sedentary behaviour results.  Sedentary behaviour is a broad-brush term used to describe sedentary time and the pattern in which that sedentary time occurs in the 24 hour day.  To say that sedentary behaviour is increased or decreased is vague and conveys no information on what has happened.  So to open the conclusions with "High levels of sedentary behaviour...." is inappropriate.  What is meant I think is sedentary time.  That should be changed.

 Moreover, what are "high levels" of sedentary time?  For that to be accurately expressed there needs to be a comparison with another population.  The sedentary time reported in the reviewed papers probably is high, but to ascertain that it is, the data should be compared with similarly measured sedentary time in an age matched population.  This is also important in saying that sedentary time is "almost epidemic" in the ID population; compared to what standard? (international guideline perhaps, or to their peers without ID; the new WHO guidelines might be used as a comparator, as are Canadian 24-hour guidelines).

 The authors have recognised the large variability in methods used to measure sedentary time and the problematic issues of comparing results from different methods; that might also be a recommendation in the conclusions; that more objective measures are used.

 The authors should carefully check the language used in describing study outcomes; an example of this is at the end of the intro in reference to the Sardinha 2015 paper, which reported on a cross sectional study.  "breaking time spent sedentary has been shown to prevent disability".  "Prevent" implies causation, and should be replaced by a phraseology that refers to correlation rather than causation.

 Overall a thorough review, with some limitations in the lack of precision in writing which should be edited.

Are the rationale for, and objectives of, the Systematic Review clearly stated?

Yes

Is the statistical analysis and its interpretation appropriate?

Yes

Are sufficient details of the methods and analysis provided to allow replication by others?

Yes

Are the conclusions drawn adequately supported by the results presented in the review?

Partly

Reviewer Expertise:

Sedentary behaviour measurement; sedentary behaviour and health.

I confirm that I have read this submission and believe that I have an appropriate level of expertise to confirm that it is of an acceptable scientific standard, however I have significant reservations, as outlined above.

# Peer review: Reviewer response for version 2

**DOI:** 10.21956/hrbopenres.14619.r30434

**Published:** 2021-09-24

**Authors:** Chris Hatton

**Affiliations:** 1 Department of Social Care and Social Work, Manchester Metropolitan University, Manchester, UK

I would like to thank the authors for their thorough consideration of my comments on the original version of this review. They have addressed my comments where relevant and have justified convincingly their reasons for not making revisions where relevant.

 I have no further comments to make on this excellent review.

Are the rationale for, and objectives of, the Systematic Review clearly stated?

Yes

Is the statistical analysis and its interpretation appropriate?

Yes

Are sufficient details of the methods and analysis provided to allow replication by others?

Yes

Are the conclusions drawn adequately supported by the results presented in the review?

Yes

Reviewer Expertise:

Health and social inequalities and people with intellectual disabilities

I confirm that I have read this submission and believe that I have an appropriate level of expertise to confirm that it is of an acceptable scientific standard.

# Peer review: Reviewer response for version 1

**DOI:** 10.21956/hrbopenres.14508.r29774

**Published:** 2021-08-11

**Authors:** Chris Hatton

**Affiliations:** 1 Department of Social Care and Social Work, Manchester Metropolitan University, Manchester, UK

The authors are to be congratulated on this well-conducted and clearly written review of an important issue. The existing literature in this area is not straightforward and the authors have taken good and defensible decisions about what to include and how to analyse the literature they have reviewed.

 I only have minor suggestions for revisions, largely around issues of presentation, below:

- 1.0. the authors need citations to accompany their assertion that people with ID now live longer than in previous decades.
- 1.1. the authors do address this later in the paper, but at this point in the introduction it might be worth having a bit more a rationale for their definition of SB, given that it includes physical inactivity which the authors at the start of this section say is very different to SB.
  At the end of this section, it seems a bit odd to say that a paucity of investigation into SB is a reason to conduct a systematic review - if this were the case then there would be nothing to review!
- 2.1. I think the sentence beginning 'A focused and well-defined question..." could be cut as readers can be assumed to know this.
  The I of the PICO implies that people in institutional or hospital settings will not be included in the review, but this isn't followed through in the inclusion or exclusion criteria.
- 2.2. The research question concerns older adults, which I'm guessing was the original intention but needs to be amended to fit the eventual search strategy?
  How was the eligibility criterion of adults aged 18+ with an intellectual disability operationalised? In systematic reviews I've been involved in, sometimes participant samples aren't wholly people with intellectual disabilities and we've had to use criteria like 75%+ of the participant sample are people with intellectual disabilities.
  Throughout the Methods section the tense needs to be checked, so it's all in past tense (the search was conducted) rather than in the future (the search will be conducted).
- 2.3.3. I think there's a "not" missing in the sentence "Those that did meet the inclusion criteria were omitted".
- Figure 1. I'm not sure this is needed. It's unclear why it's in essence the same figure repeated, and this process is described very clearly and in more detail in the text.
- 2.4. I think the short para starting "These tools are used to..." can be cut - I think readers can be assumed to be convinced of this already.
- Tables 2 and 3 - I think both of these can be cut as they add little/nothing to the text descriptions.
- 3.2. This is a decision for the editors, but I think the table summarising the 25 studies is a central component of the review and should be included in the main paper rather than as supplementary material. Hopefully, my other suggestions about potentially cutting other tables offsets this a little?
- Table 4 - I think this can be cut as all the information is already in Figure 2 and described in the text.
- Table 5 - this could be a supplementary table rather than in the main paper?
- I wonder if there is a way of incorporating Tables 7 and 8 into a more detailed Table 6?
- 4.2.3. I think the meta-analyses and the Forest Plots are really helpful, well-conducted and well-presented. Given the other Forest Plots, I was surprised not to see one on Steps per day and weekdays/weekend days. 
- 4.2.4 The formula and it's description can be cut.
- For the Forest Plots, I was not clear why they were divided by 10 or 100 - for me it made them more difficult to intuitively interpret.
- Figure 4 - as this is a Forest Plot of steps per day, is it possible to change the presentation of the Figure so that: a) it doesn't say Mean difference at the top; b) it doesn't have a minus part of the horizontal axis? (this is the same for Figure 7). 
- 4.3 - first sentence. "SB was assessed using time..." - is this time being active or time being sedentary?
- Figure 5 - screen time. Should the first study here be Melville et al rather than Carlson?
- Given there are only 3 studies, all described in the text, is Table 10 necessary?
- 5.0. The discussion is well-written and I have no comments on this section.

Are the rationale for, and objectives of, the Systematic Review clearly stated?

Yes

Is the statistical analysis and its interpretation appropriate?

Yes

Are sufficient details of the methods and analysis provided to allow replication by others?

Yes

Are the conclusions drawn adequately supported by the results presented in the review?

Yes

Reviewer Expertise:

Health and social inequalities and people with intellectual disabilities

I confirm that I have read this submission and believe that I have an appropriate level of expertise to confirm that it is of an acceptable scientific standard, however I have significant reservations, as outlined above.

# Response

**Published:** 2021-09-13

**Authors:** Louise Lynch

**Affiliations:** Trinity College Dublin, Ireland

Thank you for taking the time to review this article on the Sedentary behaviour levels of people with an intellectual disability. Your review and feedback has been very constructive and valuable. Responses to each point are included below. 

- 1.0. the authors need citations to accompany their assertion that people with ID now live longer than in previous decades.

*The reference was after the next sentence and has been moved to directly after the statement that people with ID live longer.*

- 1.1. the authors do address this later in the paper, but at this point in the introduction it might be worth having a bit more a rationale for their definition of SB, given that it includes physical inactivity which the authors at the start of this section say is very different to SB.

*Clarification has been included.*

- At the end of this section, it seems a bit odd to say that a paucity of investigation into SB is a reason to conduct a systematic review - if this were the case then there would be nothing to review!

*This has  been changed.*

- 2.1. I think the sentence beginning 'A focused and well-defined question..." could be cut as readers can be assumed to know this.
  The I of the PICO implies that people in institutional or hospital settings will not be included in the review, but this isn't followed through in the inclusion or exclusion criteria.

*Sentence removed and institutional and hospital added.*

- 2.2. The research question concerns older adults, which I'm guessing was the original intention but needs to be amended to fit the eventual search strategy?

*Older has been removed.*

- How was the eligibility criterion of adults aged 18+ with an intellectual disability operationalised? In systematic reviews I've been involved in, sometimes participant samples aren't wholly people with intellectual disabilities and we've had to use criteria like 75%+ of the participant sample are people with intellectual disabilities.

*Adults with ID aged 18+ were included in the study. This was through the article review process. If data on people with an intellectual disability was not separate the article was excluded.*

- Throughout the Methods section the tense needs to be checked, so it's all in past tense (the search was conducted) rather than in the future (the search will be conducted).

*All tenses amended*

- 2.3.3. I think there's a "not" missing in the sentence "Those that did meet the inclusion criteria were omitted".

*'Not' added in.*

- Figure 1. I'm not sure this is needed. It's unclear why it's in essence the same figure repeated, and this process is described very clearly and in more detail in the text.

*Figure deleted and added in as extended data.*

- 2.4. I think the short para starting "These tools are used to..." can be cut - I think readers can be assumed to be convinced of this already.

*Paragraph deleted*

- Tables 2 and 3 - I think both of these can be cut as they add little/nothing to the text descriptions.

*Deleted Table 3. Table 2 kept as it details the ranges used which are not detailed in the text.*

- Table 4 - I think this can be cut as all the information is already in Figure 2 and described in the text.

*Table 4 has been deleted.*

- This is a decision for the editors, but I think the table summarising the 25 studies is a central component of the review and should be included in the main paper rather than as supplementary material.

*Submitted for inclusion in text.*

- Table 5 - this could be a supplementary table rather than in the main paper?

*Moved to supplementary material.*

- I wonder if there is a way of incorporating Tables 7 and 8 into a more detailed Table 6?

*This was attempted but proved too hard to read.*

- 4.2.3. I think the meta-analyses and the Forest Plots are really helpful, well-conducted and well-presented. Given the other Forest Plots, I was surprised not to see one on Steps per day and weekdays/weekend days. 

The steps per day forest plot was Figure 4. The title has been updated to include ‘per day’

- 4.2.4 The formula and it's description can be cut.

*This has been deleted.*

- For the Forest Plots, I was not clear why they were divided by 10 or 100 - for me it made them more difficult to intuitively interpret.

*The forest plot numbers were divided by 10 or 100 so results could be seen clearly. *

- Figure 4 - as this is a Forest Plot of steps per day, is it possible to change the presentation of the Figure so that: a) it doesn't say Mean difference at the top; b) it doesn't have a minus part of the horizontal axis? (this is the same for Figure 7). 

*It is not possible to make these changes.*

- 4.3 - first sentence. "SB was assessed using time..." - is this time being active or time being sedentary?

*This is the time spent in sedentary behaviour. Text in the article has been changed to make clearer.*

- Figure 5 - screen time. Should the first study here be Melville et al rather than Carlson?

*The plot is correct as the Melville study did not give specific screen times but rather a nine-point scale which consisted of ranges. I have amended the text to include the Carlson study for clarity.*

- Given there are only 3 studies, all described in the text, is Table 10 necessary?

*Table 10 details the weekend versus weekday SB levels which are not all included in the text.*
